# Chicken-derived R-spondin1, Wnt3a, Nrg1 and human FGF1 allow branched growth of chicken intestinal organoid cultures

**DOI:** 10.1016/j.psj.2026.107340

**Published:** 2026-06-24

**Authors:** Jean Paul ten Klooster, Kitty van Summeren, Jasper de Louwere, Francisca C. Velkers, Raymond H.H. Pieters

**Affiliations:** a Innovative Testing in Life Sciences and Chemistry, Research Centre Healthy and Sustainable Living, University of Applied Sciences Utrecht, Utrecht, the Netherlands; b Department Population Health Sciences, Division Farm Animal Health, Faculty of Veterinary Medicine, Utrecht University, Utrecht, the Netherlands; c Institute for Risk Assessment Sciences, Population Health Sciences Division, Utrecht University, Utrecht, the Netherlands

**Keywords:** Organoid, Intestine, Wnt-pathway, Stem cells

## Abstract

Intestinal organoids are widely used in mammalian studies to mimic and study intestinal function and host–pathogen interactions without using of whole animal studies. For chicken, this was not yet feasible. Recently, we have shown that embryonal intestinal organoids can grow for 10-15 passages (approximately 6 weeks) when using chicken-derived Rspo1 and Wnt3. In this paper, we show that replacing Wnt3 with chicken Wnt3a and adding chicken Nrg1 and human FGF1 results in efficient and long lasting growth, over 40 weeks, of embryonal chicken intestinal organoids. Moreover, the typical branching phenotype, which is normally observed for mammalian species such as mouse and pig intestinal organoids, is now also observed in the chicken organoids when Rspo1, Wnt3a, Nrg1 and hFGF1 are used. This branching is enhanced by inhibiting FoxO1 with AS1842856, resulting in an increase of growth. Moreover, we show the presence of Lgr5 positive stem cells, goblets, endocrine cells, Paneth cells and enterocytes. In agreement with the observed presence of goblet cells, we observed mucus secretion in the 2D cultures. This new and improved culture condition for chicken intestinal organoids allows for in vitro research that can partially replace animal testing with respect to, for instance, replacement of antibiotics, feed additives and drug development.

## Introduction

Poultry meat and eggs are an important source of animal protein and essential for feeding the growing world population (McNeill et al., 2017; Réhault-Godbert et al., 2019). With the annual increase of poultry farms there is also an increase of infectious disease which are controlled by using antimicrobial drugs. To reduce the risks of antimicrobial resistance for public health, the use of antimicrobial agents for livestock farming in the European Union are nowadays under strict regulations (O’Neill, 2016). However, there is still a need to reduce and prevent intestinal diseases, which has led to a continuous search for alternatives with e.g. antimicrobial, anti-inflammatory, antioxidant, or immune-fitness promoting properties (Levy and Bonnie, 2004; Stuart and Marshall, 2004; Zhu et al., 2021).

According to European regulations (European Guideline 2010/63/EU), the use of animal testing needs to be reduced. In our lab we develop in vitro models to reduce the use of animal testing (Klooster et al., 2021; Oost et al., 2022). To study the effects of food additives and pharmaceuticals on intestinal health of chicken in vitro, we are eager to develop and culture chicken intestinal organoids that represent many physiological functions of the intestine. Recently, we showed that by replacing the mammalian stem cell factors Rspo1 and Wnt3 with the chicken variants, embryonal intestinal organoids from chicken can be cultured for multiple passages (Oost et al., 2022). However, the growth of these organoids was limited to a max of 15 passages and did not show the typical branched morphology normally seen in mammalian species (Oost et al., 2022). Although other papers show isolation and cultures of chicken intestinal organoids, none of these papers showed long-term cultures with more than 10 passages(Mitchell et al., 2024)(Kang and Lee, 2024; Li et al., 2018; Mitchell et al., 2024; Shin et al., 2025; Wang et al., 2022; Zhao et al., 2022). However, these papers do nicely show the usefulness of chicken organoids in fundamental and applied research with the potential of being used as largescale screening tools for feed and pharma.

Here we show that chicken organoids grown in the presence of the chicken stem cell factors Rspo1 and Wnt3a, combined with chicken Nrg1 peptide and human FGF1 were able to grow rapidly for over 40 passages, with the typical branched phenotype so familiar for intestinal organoids of mammalian species (Sato and Clevers, 2013). In addition, they maintain their ability to produce mucus, chromogranin A and serotonin, suggesting that goblets and endocrine cells are present. Moreover, based on cell phenotype in 2D cultures, we also observed outgrowth of enterocytes, indicating that these cultures can be used for absorption and metabolism of nutrients and chemicals.

## Methods

### Medium and reagents

Basic culture medium (BCM): Advanced Dulbecco's Modified Eagle Medium/Ham's F-12 (advanced DMEM/F12) supplemented with 10% Fetal Calf Serum (FCS), 1% GlutaMAX, 2.5 ml penicillin-streptomycin 10,000 U/mL (all Gibco, Life Technologies Limited, Paisley, UK).

Chicken organoid culture medium (COCM): Basic Culture medium, supplemented with:

CHIR99021 2 µg/ml (Sigma-Aldrich, Saint Louis, MO, USA). valproic acid 0.78 mM (Sigma-Aldrich, Saint Louis, MO, USA).

DMH-1, a bone morphogenetic protein-4 (BMP4) inhibitor, 0.5 µg/ml (Sigma-Aldrich).

0.5 µM A83 (Sigma, SML0788).

FoxO1 inhibitor, AS1842856, 0.2 µM (Sigma-Aldrich).

Chicken-specific Wnt3a, R-spondin1 and Nrg1 (1:5, in-house production).

100 ng/ml FGF1 (Peprotech, 100-17A).

### In house production of chicken-specific growth factors Wnt3a/R-spondin1/Nrg1

Stably expressing Human Embryonic Kidney (HEK293t) cells were produced, to get chicken-specific growth factors Wnt3a, Rspo1 and Nrg1. Chicken Rspo1-DYK (Genscript; OGa18707), Wnt3a-DYK (Genscript; OGa25549D) and Nrg1 (Genscript OGa152948; Fig. S1) cDNA clones were subcloned into Tol2-containing E4 plasmid (Kind gift from Stefan Schulte-Merker). Tol2 sequences allowed us to have stable genomic integration of these genes in Hek293t cells upon Puromycin selection (Invitrogen; ant-pr-1). After selection, Hek293t cells co-expressing Rspo1, Wnt3a and Nrg1 were expanded in culture medium and when cells reached 100% confluent for 2 days, supernatant was collected and centrifuged for 5 minutes at 335 Xg. For organoid cultures, these supernatants were diluted 10 times in COCM.

### Isolation of avian intestinal stem cell containing structures and development of 3D intestinal organoids

Embryonic day 18 (ED18) Lohmann Brown chicken eggs (Gallus gallus) were obtained from the Department of Farm Animal Health, Faculty of Veterinary Medicine, Utrecht University, the Netherlands. Both small and large intestines were collected from the embryos post mortem. This method was conducted in accordance with protocols approved by the Department of Biomolecular Health Sciences, Division of Infectious Diseases & Immunology of the Utrecht University. Although according to European legislation for the protection of animals used for scientific purposes (Directive 2010/63/EU) experiments with embryonated chicken eggs are not considered animal experiments, the study was carried out in compliance with the ARRIVE guidelines. Immediately after collection, the intestines were placed in a petri dish containing 10 mL of 5 mM ice-cold phosphate-buffered saline-ethylenediaminetetraacetic acid (PBS-EDTA; Lonza, Basel, Switzerland). After washing with PBS-EDTA, the intestines were cut into small segments and placed into a 50 mL conical tube containing PBS-EDTA. To get a homogenous suspension of intestinal stem cell containing structures, 2 mL of PBS-EDTA containing intestinal segments was minced using a potter-elvehjem PTFE pestle and glass tube (Sigma-Aldrich). The intestinal suspension was collected in a 15 ml tube and centrifuged at 335 Xg for 5 min at 4°C. After centrifugation, the supernatant was discarded and aggregates were resuspended in 10 mL PBS-EDTA and centrifuged again. This was repeated until supernatant became clear. Next, the suspension was filtered through a 100-µm cell strainer (Corning, Amsterdam, the Netherlands) to get a uniform intestinal solution. The filtrate was centrifuged at 335 Xg for 5 min at 4°C and supernatant was discarded. The pellet was resuspended in 800 µL of ice-cold Matrigel (Corning; 354234). Two drops of 25 µL intestinal stem cell containing matrigel suspension were added per well in a pre-warmed 12 well culture plate (Corning). Following polymerization of Matrigel, 2 mL of COCM was added per well and the culture plate was placed at 41°C, 5% CO2. 3D intestinal organoids were passaged twice a week in a 1:6 ratio, by dissociating the organoids embedded Matrigel domes in 1 mL fresh BCM through vigorous pipetting. After dissociation, the organoid solution was centrifuged at 335 Xg for 5 min at 4°C. Supernatant was discarded and the organoids pellet was suspended in ice-cold Matrigel for seeding to expand the 3D intestinal organoids culture. The average number per dome is 30-50 organoids. See supplementary Fig. S2 for a visual abstract of this method.

### Conversion of 3D intestinal organoids to monolayer culture

Prior to seeding the chicken intestinal organoids, 12 mm sterile glass coverslips (Waldemar Knittel Glasbearbeitungs GmbH, Brunswick, Germany) were coated with Matrigel. This was done by placing the coverslips in a 24 well culture plate, and 40 µL of 2.5% ice-cold Matrigel solution in Dulbecco’s Phosphate Buffered Saline with calcium and magnesium (DPBS+/+) was added per well, followed by a 2 h incubation at 37°C. The Matrigel solution was removed by pipetting and replaced with 2 ml COCM medium.

To obtain an organoid suspension, 3D intestinal organoids embedded in the Matrigel matrix were dissociated mechanically by vigorous pipetting using a 1000-µL pipet in BCM. A total of 6 domes were pooled and the solution was centrifuged at 335 Xg, 5 min at 4°C. Supernatant was discarded and the pellet was resuspended in 600 µL culture medium and 100 µL was seeded on 6 matrigel covered glass coverslips resulting in approximately 30-50 organoids per coverslip. Then the culture plate was placed at 37°C, 5% CO2. Intestinal organoid monolayers that were formed on glass coverslips covered almost 70% of the coverslips within 2 days.

### Immunohistochemical staining

To identify various chicken intestinal epithelial cell lineages; glass coverslips containing the monolayer cultures were fixed with 200 µL of DPBS+/+ and 4% paraformaldehyde (PFA) solution for 1 h at RT. Later on, the monolayer culture was washed with 1 mL of DPBS-/- and 10 mM glycine (Merck Millipore, Burlington, MA, USA) solution to quench the fixative. Following fixation, the monolayer culture was blocked in 300 µL of blocking buffer consisting of DPBS-/-, 0.05% Tween-20 (Sigma-Aldrich), and 2% bovine serum albumin (Sigma-Aldrich) for 1 h at 25°C. Coverslips were then stained with monolayer faced down on parafilm containing 25 µL of blocking buffer containing primary antibodies, as mentioned in Table 1, overnight at 4°C. The next day, monolayer cultures were stained with secondary antibodies as mentioned in Table 1 for 1 h at RT. As a negative control we used the secondary antibody only. In addition to that, actin and nuclei were stained for 1 h at RT using Rhodamine Phalloidin (Life Technologies) and Hoechst 33342 (Sigma-Aldrich; 14533), respectively. Images were acquired on a Olympus IX71 fluorescence microscope using CellSens software (Olympus Corporation).

**Table 1:** Characteristic of the used antibodies.

Antibody	Concentration	Species cross-reactivity	Target site	Host/Isotype
Anti-Sox9 AB5535 Sigma-Aldrich	1:500	Human, Mouse, Rat, Chicken	marker for stem/progenitor cells	Rabbit
Chromogranin-A Immunostar 20086	1:500	Buffalo, Chicken, Cow, Dolphin, Human, Mouse	Enteroendocrine cells	Rabbit
Muc5AC (clone 45M1) Abcam Ab212636	1:500	Chicken, Dog, Human, Mouse, Rabbit, Rat	Goblet cells	Rabbit
Donkey anti-Rabbit IgG (H+L) Highly Cross-Adsorbed Secondary Antibody, Alexa Fluor 488	1:1000	Rabbit	Gamma Immunoglobins Heavy and Light chains	Donkey/IgG

### Enzym-linked lectin assay (ELLA)

To evaluate mucin production in cell cultures, an enzyme-linked lectin assay was used to quantify secreted mucins. Post-incubation, medium samples were collected from each well and stored at −20°C. High-binding plates (Maxisorp™; VWR, 735-0083) were coated with 4 µg/mL wheat germ agglutinin (WGA; Sigma-Aldrich, 61767) for 2 hours at 37°C. Wells were subsequently washed four times with PBST (0.05% Tween-20 in PBS) and blocked with PBST for 1 hour at 37°C. After blocking, wells were tapped dry without additional washing.

ODM culture medium samples and porcine mucin standards (Sigma-Aldrich, M2378) ranging from 0.156 to 10 µg/mL were added and incubated for 1 hour at 37°C. After incubation, wells were washed four times with PBST (0.05%) prior to incubation with 2 µg/mL horseradish peroxidase-labeled soybean agglutinin (Sigma-Aldrich, Saint Louis, L2650) for 1 hour at 37°C. Following incubation, wells were washed four times with PBST (0.05%). TMB substrate was then added for five minutes in the dark at room temperature, and the reaction was stopped with 1 M H2SO4. Absorbance at 450 nm (A450) was measured using a BMG FLUOstar Omega. A culture medium control was utilized to determine the signal-to-background ratio.

### Real‑time quantitative PCR

The gene expression level of various passages of 3D and 2D intestinal organoids was determined by Real-Time quantitative PCR (RT-qPCR). Two wells of 3D and 2D intestinal organoids culture were harvested from Matrigel using cold Advanced DMEM/F12 medium by vigorous pipetting and centrifuged at 295 × g, 5 min at 4°C. The pellet was lysed in RLT buffer (Qiagen, Venlo, the Netherlands) supplemented with β-mercaptoethanol (Sigma-Aldrich). Total RNA was extracted using RNeasy mini kit (Qiagen, 74106) according to manufacturer’s instructions and RNA was quantified using NanoDrop spectrophotometer (Isogen, de Meern, The Netherlands). Reverse transcription of 0.5 μg of total RNA was done using iScript cDNA synthesis kit (Bio-Rad, 170-8891) as per manufacturer’s instructions and cDNA was diluted 1:1 ratio in milli-Q. RT-qPCR assays were performed using CFX384 real-time PCR detection system (Bio-Rad). Primer LGR5 (Forward: CCC ACT GCT ATC AGGACA CTAAC; Reverse: GAG GCA CCA TTT AAA GTC AGAG) MUC2 (Forward: TGG TCT CTG TGG GGA TTA CA; Reverse: CCG AGT TTC ATC AGG GTC TT) was used as a marker for respectively stem cells and goblets. Normalization of LGR5 and Muc2 was done using GAPDH (Forward: GTG GTG CTA AGC GTG TTA TC; Reverse: GCA TGG ACA GTG GTC ATA AG) as the housekeeping gene. 5 μL of cDNA template was added per well consisting of 20 μL reaction mixture using 12.5 μL SYBR Green Supermix, 5.5 μL of distilled water, 1 μL (400 nM) of forward and reserve primers. PCR thermal cycle conditions used were as follows: an initial 5 min step at 95°C, followed by 40 cycles of 92°C for 10 s, 55°C for 10 s, and 72°C for 30 s. This was followed by cycle 3 consisting of 95°C for 1 min, and 65°C for 2 min.

### RNA sequencing

RNA sequencing was performed by Novogene. Total RNA was extracted using RNeasy mini kit (Qiagen, 74106) according to manufacturer’s instructions and RNA was quantified using NanoDrop spectrophotometer (Isogen, de Meern, The Netherlands). All samples were found to be of acceptable quality for sequencing (RIN > 9.0) and sent to Novogene (UK) for sequencing by poly-A capture, library preparation and analysis on the Illumina Novaseq using 150 bp paired end sequencing. Data used are fpkm values provided by Novogene.

**Data analysis.** Significant differences in the branching of organoids when culturing with different supplements, and the mRNA levels of Lgr5 and Muc2 between multiple passage numbers, were analyzed by the Kruskal–Wallis test, followed by the Dunn’s multiple comparisons test in Graphpad Prism (version 9.3.1, San Diego, CA, USA). A P-value < 0.05 as considered significant.

## Results

### Chicken-derived growth factors Rspo1, Wnt3a and Nrg1, combined with human FGF1 are preferred for the maintenance of chicken intestinal organoids

**Fig. 1:**
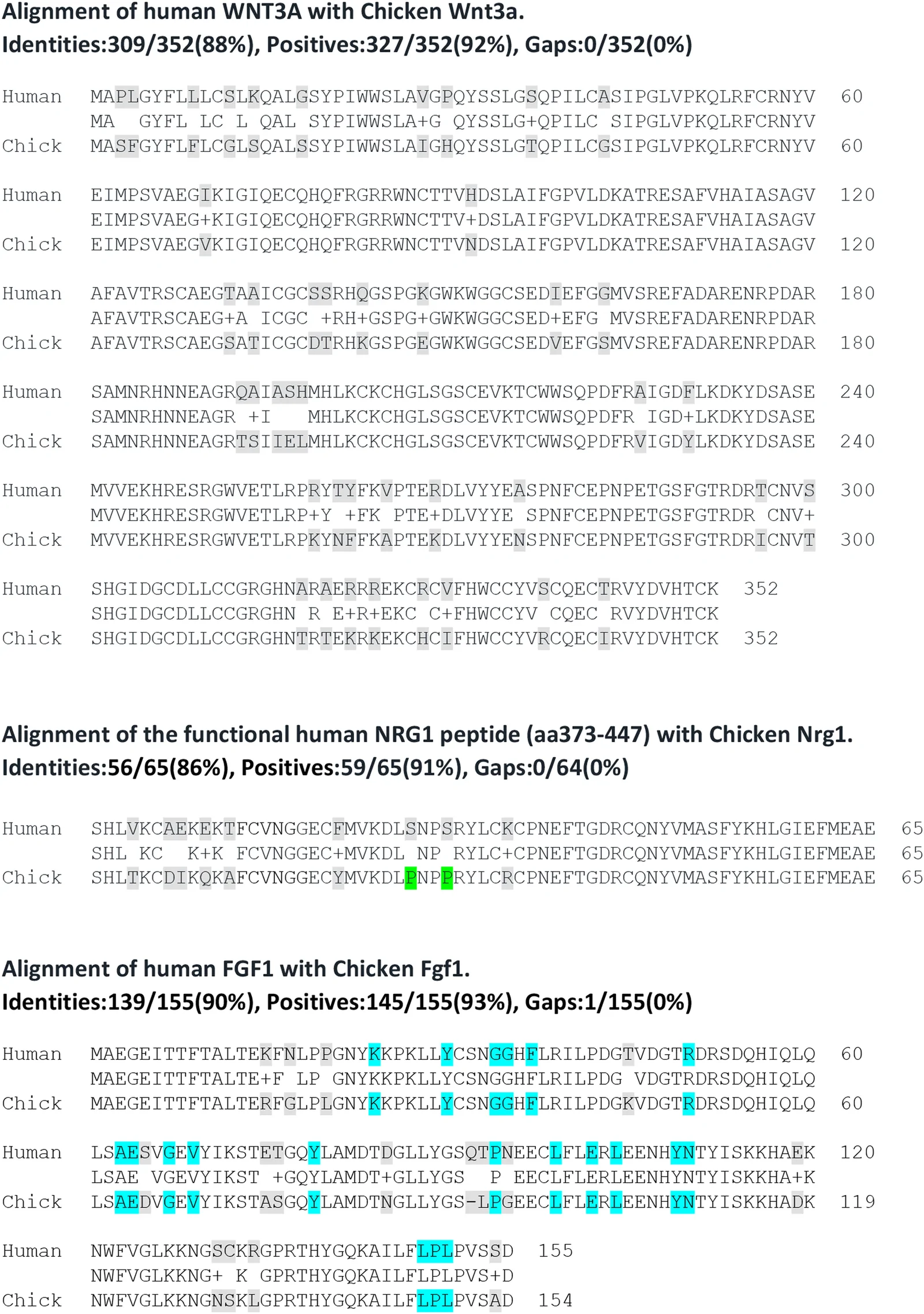
Alignment of human and chicken Wnt3a, Nrg1-peptide and Fgf1. The amino acid sequences of human and chicken Wnt3a, Nrg1 and Fgf1 were aligned by using BLAST. Percentage of similarity and gaps are indicated. Gray highlighted amino acids indicate differences between the human and chicken sequences. Green marked prolines are highlighted because of their potential importance as helix breakers. Blue highlighted amino acids in FGF1 are involved in the interaction of FGF1 with its receptor.

Previously, we have shown that embryonal chicken organoids could grow in the presence of chicken Rspo1 and Wnt3, however, growth could not be maintained beyond passage 15 (Oost et al., 2022). For mammalian organoid cultures most described protocols use Wnt3a instead of Wnt39,10. However, we showed that human WNT3A did not support growth of chicken organoids (Oost et al., 2022), which could be easily explained by the low homology between human WNT3A and chicken Wnt3a (Fig. 1). To test whether chicken Wnt3a would enhance the growth of chicken intestinal organoids we cloned chicken Wnt3a into the Tol2-containg E4 vector.

In addition to chicken Wnt3a, we also explored other growth factors such as Nrg1 and FGF1. Nrg1 is a protein of 841 aa, however, a truncated Nrg1 peptide (aa 373-447) is fully active and often used to increase mammalian cell growth (Jardé et al., 2020). Therefore, we were interested if a chicken variant of this peptide would increase the growth of chicken organoids. Alignment of this peptide between human and chicken amino acid sequence shows homology of 86% (Fig. 1).

FGF1 is essential for proper growth of organoids and it was shown that it is produced by mouse organoids themselves (Boonekamp et al., 2019). We do not know if chicken intestinal organoids can produce FGF1 and therefore we decided to include this factor into our cultures. Based on the high homology between chicken and human FGF1 (Fig. 1), we first tested a commercially available recombinant human FGF1 peptide on the growth of chicken intestinal organoids.

**Fig. 2:**
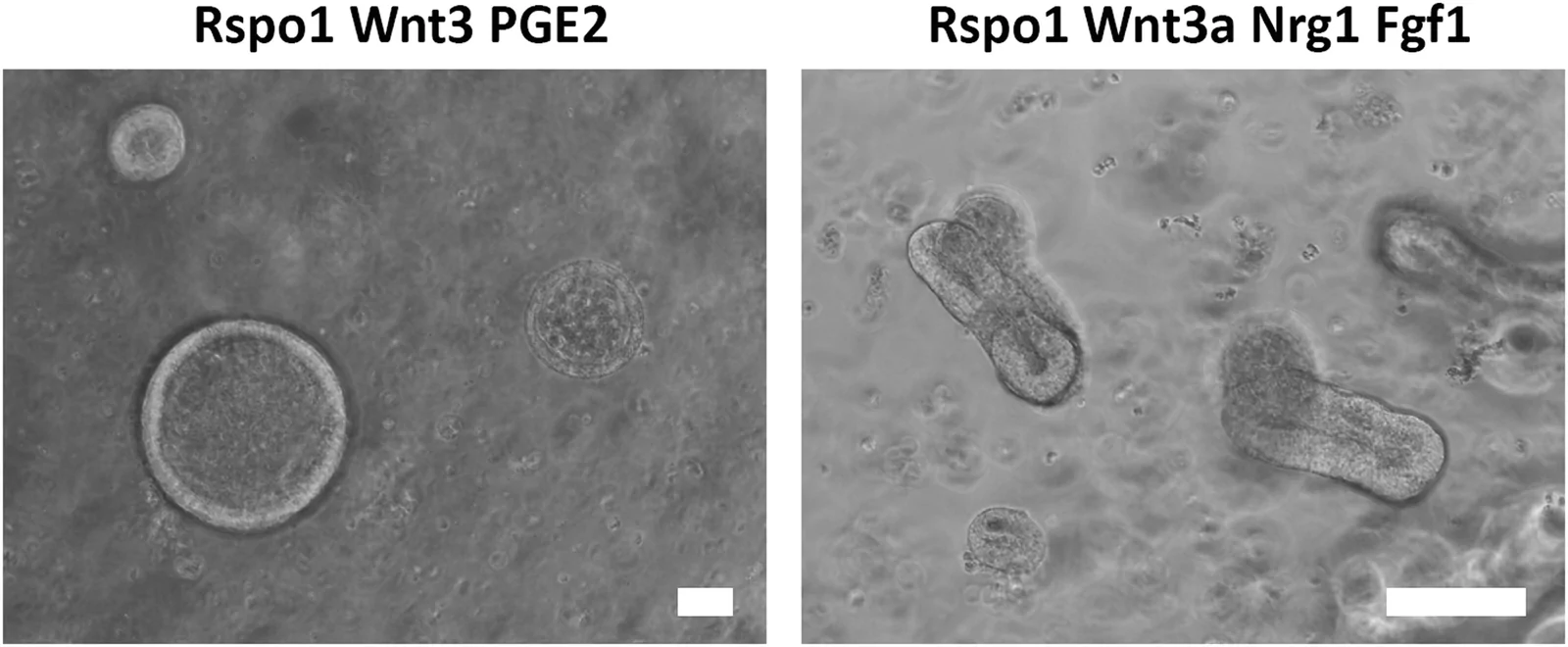
Chicken Wnt3a and Nrg1, combined with human FGF1 shows better growth of chicken embryonal intestinal organoids. Chicken embryonal intestinal organoids were allowed to grow in Matrigel droplets for 3 days in either complete organoid medium supplemented with chicken Rspo1, Wnt3 and PGE2 or Rspo1, Wnt3a, Nrg1-peptide and human FGF1. White bars indicate a size of 100 μm.

To reduce nutrient consumption of Hek293t cell-derived Rspo1, Wnt3a and Nrg1 containing supernatants, we made a clone containing all three genes, thus allowing us to use only 10-20% of supernatant instead of 30-60%. In addition to these in-house produced growth factors and the human recombinant FGF1 we also included the standard organoid culture compounds previously described (Oost et al., 2022) DMH1 (BMP4 inhibitor), valproic Acid (HDAC inhibitor), Chir99021 (GSK3β inhibitor) and A83 (ALK4, 5 and 7 inhibitor). Organoids were seeded in 25 μl Matrigel droplets and allowed to grow for approximately 1 week (Fig. 2). Interestingly, our former culture conditions showed large spheroids (Oost et al., 2022), however, with this new cocktail of growth factors we observed a more compact and mildly branched phenotype which is more similar to mouse organoids (Sato and Clevers, 2013).

### The presence of FoxO1-inhibitor AS1842856 enhances early branching of chicken intestinal organoids

**Fig. 3:**
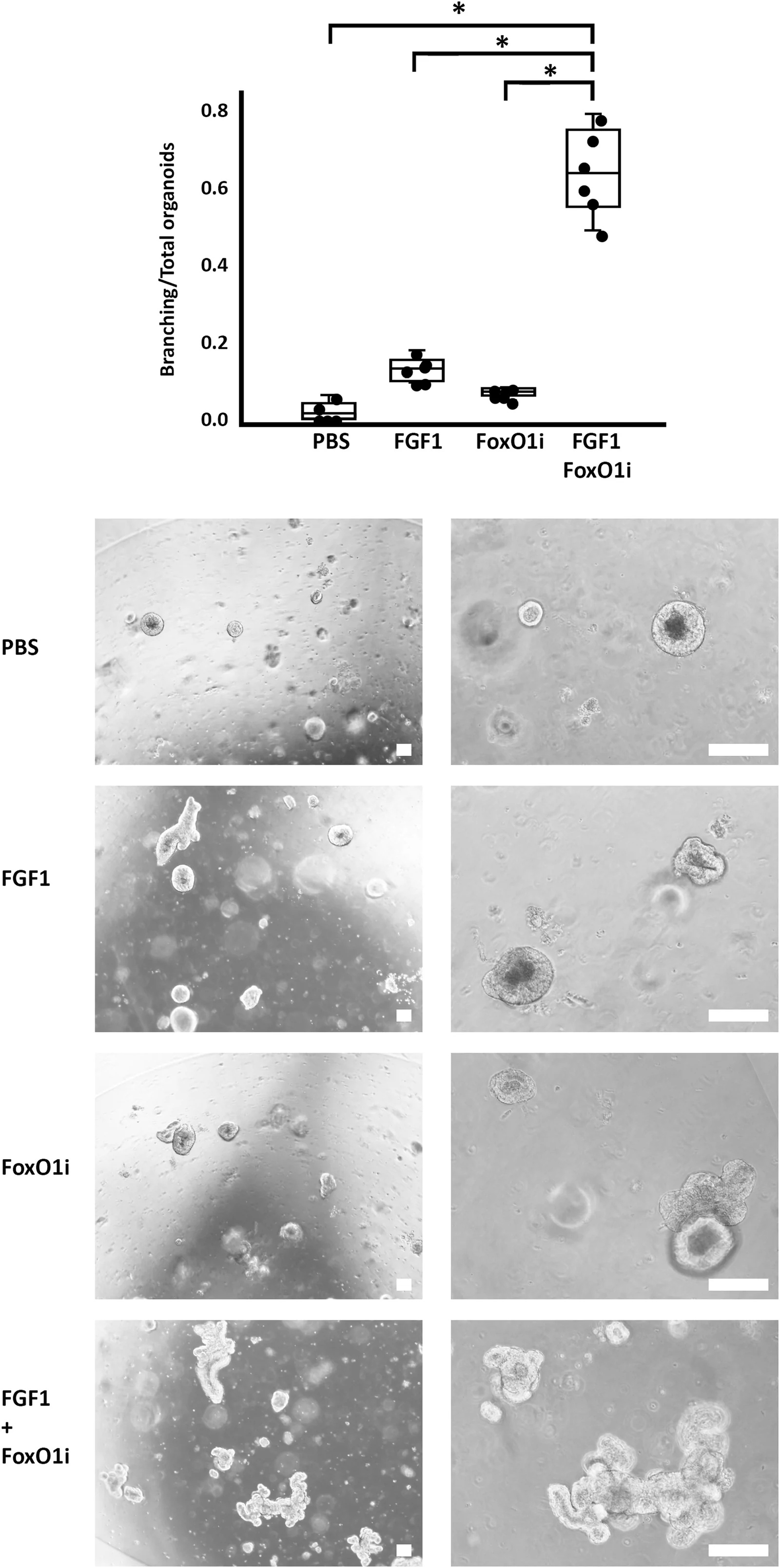
Chicken Wnt3a and Nrg1, combined with human FGF1 and the FoxO1 inhibitor AS1842856 shows increased branching of chicken embryonal intestinal organoids. Chicken embryonal intestinal organoids were allowed to grow in Matrigel droplets for 3 days in either complete organoid medium supplemented with chicken Rspo1, Wnt3a, Nrg1-peptide; human FGF1; AS1842856 as indicated. White bars indicate a size of 50 μm. The error bars represent the standard deviation of the mean. Statistics were performed using a Kruskal–Wallis test followed by Dunn’s multiple comparisons test. *p < 0.05.

Besides the growth factors used in our new medium, IGF1 might also be important to add (Fujii et al., 2018). One function of IGF1 is to inhibit the transcription factor FoxO1 14 and in our previous study, we already showed enhanced growth when the FoxO1 inhibitor AS1842856 was included in the organoid cultures of chicken organoids (Oost et al., 2022). To test if the inhibition of FoxO1 would enhance the growth of chicken intestinal organoids, we seeded organoids in Matrigel droplets in the presence of organoid medium with the FoxO1-inhibitor AS1842856 at a concentration of 0.2 µM (Fig. 3) and growth was monitored for 4 days. The addition of the FoxO1-inhibitor increased the number of branched organoids and subsequent growth, especially in the presence of hFGF1 (Fig. 3).

### Long term growth of embryonal chicken intestinal organoids in the presence of Rspo1, Wnt3a, Nrg1, human FGF1 and AS1842856

**Fig. 4:**
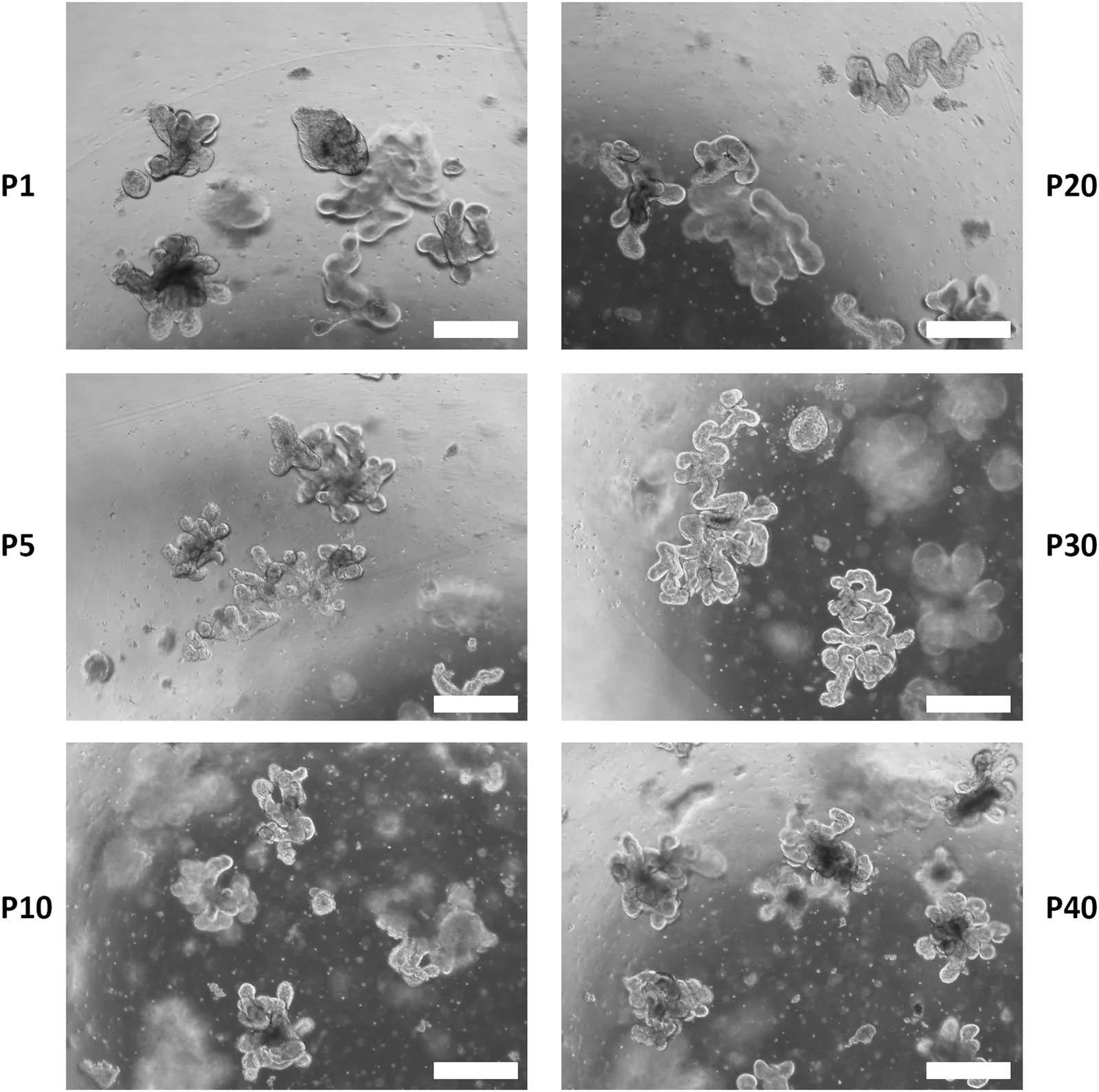
Embryonal chicken intestinal organoids grow for more than 40 passages when using basic organoid medium supplemented with Rspo1, Wnt3a, Nrg1-peptide, Fgf1 and AS1842856. Chicken embryonal organoids were grown in basic organoid medium supplemented with chicken Rspo1, Wnt3a, Nrg1-peptide, human FGF1 and AS1842856 and passaged every 4-5 days for over 40 passages. Pictures represent passage 1, 5, 10, 20, 30 and 40. White bars indicate a size of 400 mm.

In the past, we were successful at culturing embryonal-derived intestinal chicken organoids for a max of 15 passages (Oost et al., 2022), however, it would be desirable to be able to culture intestinal organoids for a longer time without the loss of function. Therefore, we cultured these organoids for multiple passages and were able to do so for at least 40 passages (Fig. 4). Indicating that we have established a very well working new protocol for culturing embryonal chicken intestinal organoids by using the chicken-derived growth factors Rspo1, Wnt3a and Nrg1, combined with human FGF1 and the FoxO1-inhibitor AS1842856.

### Chicken intestinal organoids maintain their ability to form goblet cells, endocrine cells and enterocytes

**Fig. 5:**
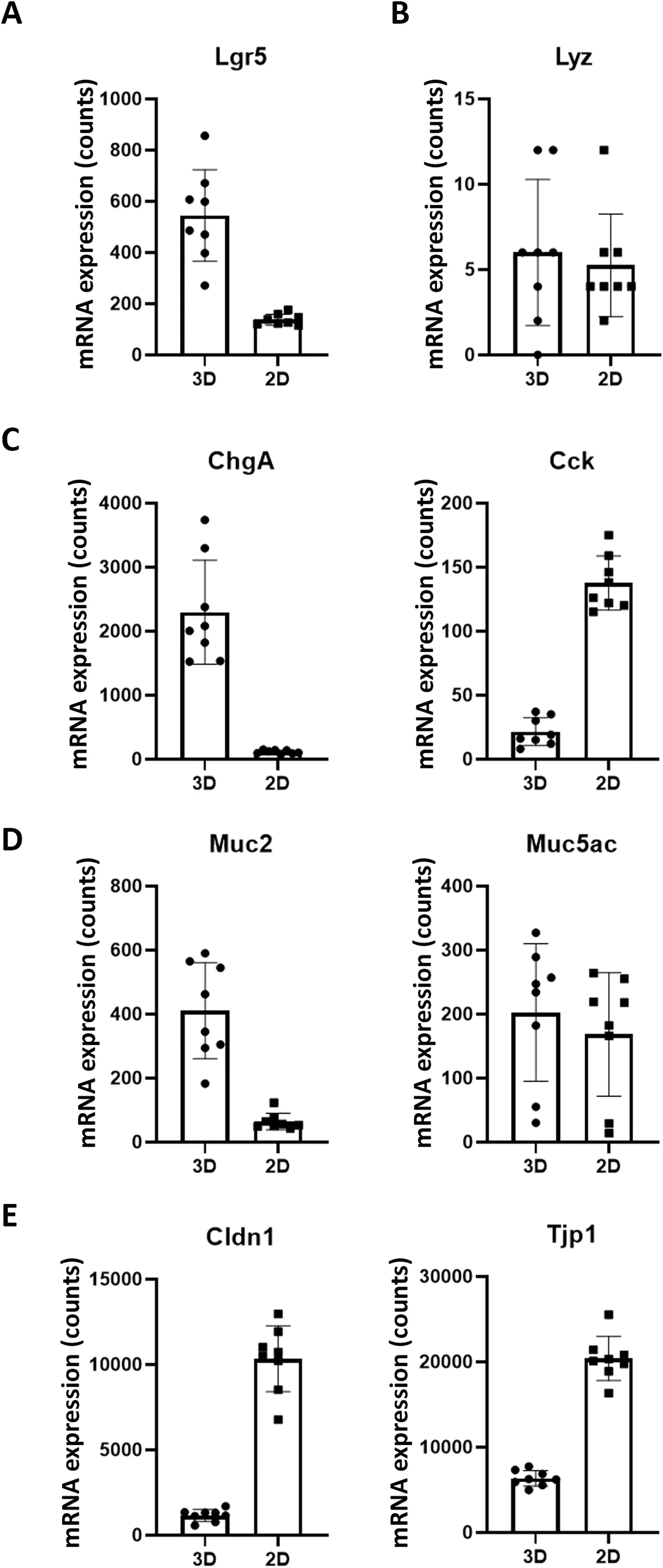
2D cultures of embryonal chicken intestinal organoids maintain their capacity to produce mucus, serotonin and Chromogranin A. mRNA expression as determined by RNA seq. A) Lgr5 as a marker for stem cells, B) Lyz as a marker for paneth cells, C) ChgA and Cck as markers for enteroendocrine cells, D) Muc2 and Muc5ac as markers for goblets and E) Cldn1 and Tjp1 as markers for enterocytes.

Stem cell-based organoids are popular because they maintain their capacity to form all intestinal cell types normally observed in vivo (Sato and Clevers, 2013, 2013; Fujii et al., 2018). To test if our cultures can still do this after 40 passages, we seeded cells in 2D on Matrigel-coated 24 wells plates and in 3D. After 4 days we collected the cells and isolated their mRNA which was used for RNA seq analyses. From these data it is clear that the 3D and 2D cultures express specific markers for stem cells (Lgr5), goblet cells (Muc5ac and Muc2), endocrine (ChgA, Cck) cells, Paneth cells (Lyz) and enterocytes (Cldn1 and Tjp2) (Fig. 5). These data also suggests that in 2D there are more enterocytes present when compared to the 3D cultures and vice versa, there are more stem cells present in 3D compared to 2D cultures.

**Fig. 6:**
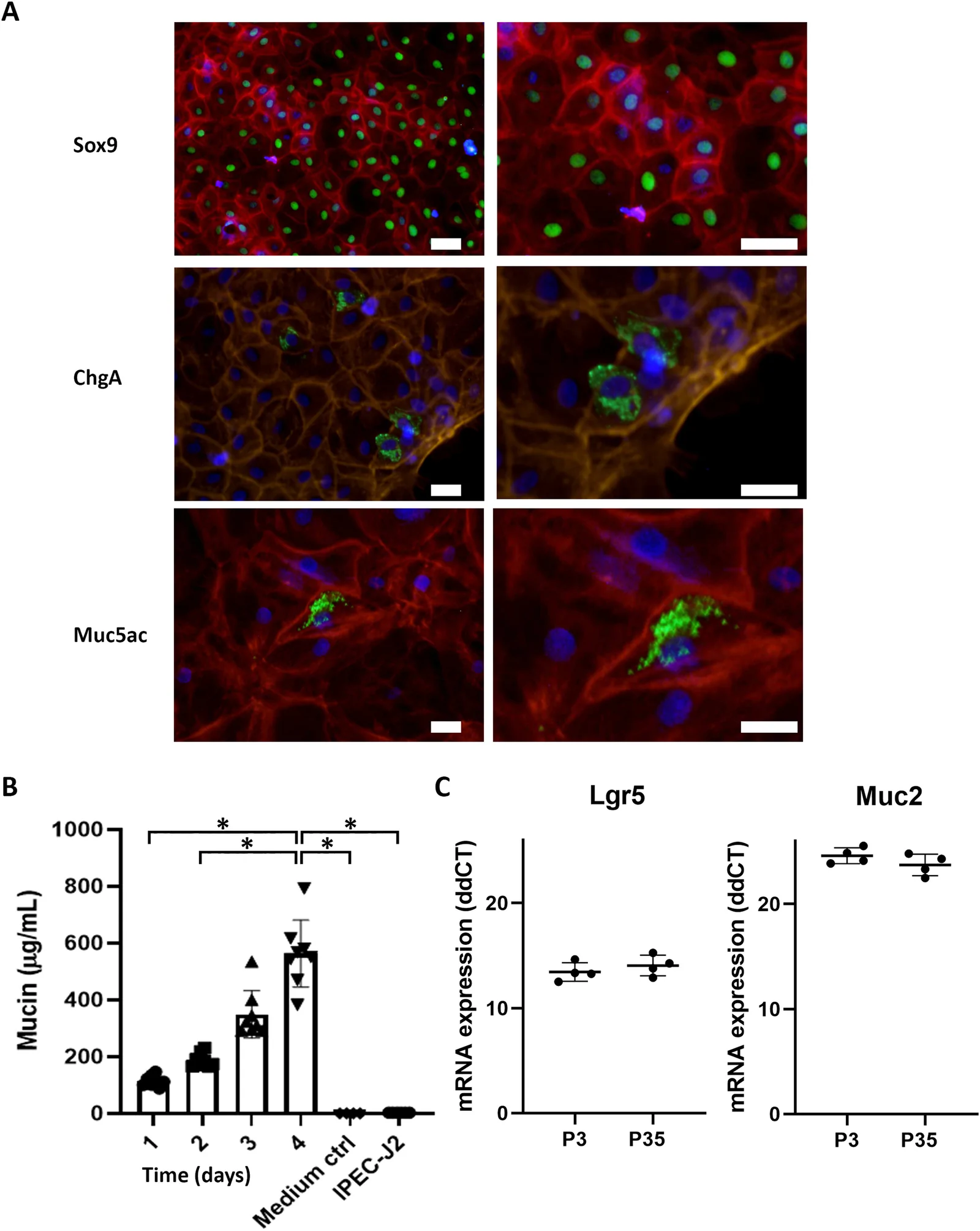
2D cultures of embryonal chicken intestinal organoids maintain their capacity to produce mucus, serotonin and Chromogranin A. A) 2D cultures of chicken embryonal and intestinal organoids were grown for 4 days. Fixed cells were stained for Sox9, ChgA, Muc5ac, F-actin and Hoechst. White bars indicate a size of 10 mm. B) Mucus secretion by 2D chicken organoids over time (days). The error bars represent the standard deviation of the mean. Statistics were performed using a Kruskal–Wallis test followed by Dunn’s multiple comparisons test. *p < 0.05. C) QPCR data on P3 and P35 for Lgr5 and Muc2 expression of 3D grown organoids. The error bars represent the standard deviation of the mean. Statistics were performed using a Kruskal–Wallis test followed by Dunn’s multiple comparisons test.

To confirm the presence of the different cell types, we seeded chicken organoids in 2D and allowed them to grow for 4 days. After fixation, we stained them for Sox9 (stem/progenitor cells), ChgA (endocrine), Muc5ac (goblet). Microscopical analyses showed the presence of Sox9, ChgA and Muc5ac positive cells (Fig. 6A). Moreover, actin staining suggests the presence of large enterocytes (Fig. 6A), which is in agreement with the mRNA expression (Fig. 5). In all, we found presence of stem/progenitor cells, endocrine cells, goblets and enterocytes in our embryonal chicken intestinal organoid cultures.

The observation that Muc5ac positive cells are present suggests the presence of mucus producing goblets. To further test this, we collected supernatants of 2D cultured organoids during 4 days and tested the mucus production by using an ELLA assay. As negative control we also took supernatants along of non-mucus producing intestinal epithelial cells from pig (IPEC-J2). As can be seen in Fig. 6B there is a clear increase of mucus during this period, confirming that these chicken intestinal organoids contain goblet cells capable of producing a functional mucus layer.

To test whether the expression of Lgr5 and Muc2 is stable over time even after later passages, we compared the expression of these 2 markers by QPCR of passage 3 and passage 35. In both cases we used 3D organoids that were allowed to grow for 4 days prior to isolating their RNA. Indeed, the expression of both markers don’t change over time (Fig. 6C), suggesting that the number of stem cells and goblets are very stable over time.

## Discussion

Here, we show for the first time that by combining chicken Rspo1, chicken Wnt3a, chicken Nrg1, human FGF1 and the FoxO1-inhibitor AS1842856 during culture of intestinal stem cells, we were able to successfully culture embryonal chicken intestinal organoids for over 40 passages, while maintaining their capacity to produce intestinal-specific cell types such as goblet cells, endocrine cells and enterocytes.

Although many other papers have worked with chicken intestinal organoids, none of these papers show that these organoids can grow for more the 3-4 weeks(Kang and Lee, 2024; Li et al., 2018; Mitchell et al., 2024; Shin et al., 2025; Wang et al., 2022; Zhao et al., 2022). The most likely explanation for this is the fact that all these papers are using mammalian growth factors such as RSPO1, WNT3A and EGF. In our previous paper we already showed that human RSPO1 and WNT3A were not able to support the growth of our chicken intestinal organoids (Oost et al., 2022). To our knowledge, we are the first to show that embryonal chicken intestinal organoids can be cultured for over 40 passages, thus allowing more controlled in vitro conditions and reduction of animals needed to obtain organoids.

Recently, a paper was published in which chicken-derived iPSCs were used to generate organoids containing neuron-like cells, although these organoids were ill-defined, it does open up the possibility of generating organ-specific organoids, such as intestinal organoids, in the near future 15. An additional paper, suggests the use of chicken organoids for testing compounds, such as butyrate, and their effect on salmonella invasion, however, they are not actually culturing these organoids but are isolating fresh material from chickens on a weekly basis (Mitchell et al., 2024). These studies do, however, nicely demonstrate the usefulness of chicken intestinal-like organoids with respect to compound testing and Salmonella invasion into enterocytes. In addition, in these papers they nicely show the use of inside-out organoids. For mammalian organoids it has been shown that normally grown organoids in Matrigel can be easily collected and transformed into inside-out organoids, suggesting that with our developed chicken organoids, we should be able to do the same, thus increasing the possibility of using chicken organoids as a research tool.

In vivo, the number of goblet cells ranges between 4 and 12%, however, this is based on Muc2 positive goblets, whereas we used Muc5ac as a marker and observed approximately 2% Muc5ac positive goblet cells in the 2D cultures. According to Tetsuya Tsukamoto (Tsukamoto et al., 2004), not all intestinal goblets express Muc5ac, but rather Muc2, suggesting that our Muc5ac stainings are underrepresenting the number of goblets in our culture, which will most likely be higher than the observed 2% that we see. In addition, organoid composition, with respect to the different cell-types, can be modified be using different growth factors or pharmaceuticals (Yang et al., 2025).

In this paper, we use embryonal material and although we have isolated crypts successfully from adult chicken intestine, which formed organoids, we were not successful at growing and passaging these adult organoids under the same condition used for the embryonal organoids (data not shown). This indicates that adult chicken organoids require, most likely, additional growth factors which are not required for embryonal chicken organoids. Although we have tested already some obvious candidates, such as chicken EGF, IGF and HGF, we were still not able to maintain adult chicken organoids for longer than 3 weeks.

In our growth medium we use chNrg1, although we tested human Nrg1 as well (data not shown) the chicken organoids grew only in the presence of the chicken variant. One possible explanation for this might be the presence of 3 prolines that are present in human NRG1 and not in chicken Nrg1. Prolines acts as a potent structural "helix breaker" and thus disrupt the 3D structure that is required for binding to its receptor (Li et al., 1996).

Now that we have established a new culture system that allows the long term culture of chicken intestinal organoids, we can start testing different aspects of chicken intestinal health in vitro, such as growth, compounds absorption and secretion, Salmonella invasion, mucus production and immunological responses. In addition, more fundamental research questions can now be answered by introducing genetic modifications in these organoids. This way we can reduce animal testing and increase fundamental research on intestinal health in chicken.

## Author contributions

J.P.t.K, contributed to the design of the experiments and performed most of the experiments, together with K.v.S, on the cultures of the chicken intestinal organoidsexperiments; In addition, J.P.t.K wrote the manuscript and made most of the figures. K.v.S cultured and analyzed the development of the chicken organoids.; J.d.L collected the intestinal fragments and assisted isolating the crypts used for culturing the organoids from chicken that were used in this paper and wrote part of the material and methods. F.V. has written the parts of the manuscript that were related to chicken and she was involved in experimental design with respect to crypt isolation. All authors reviewed the manuscript; R.P supervised the work at the HU. All authors have read and agreed to the published version of the manuscript.

## Funding

This research was performed in the public-private partnership.

## Disclosures

The authors declare that they have no known competing financial interests or personal relationships that could have appeared to influence the work reported in this paper.

## Footnotes

Supplementary material associated with this article can be found, in the online version, at doi:10.1016/j.psj.2026.107340.
