# Supplementary material for: Chicken-derived R-spondin1, Wnt3a, Nrg1 and human FGF1 allow branched growth of chicken intestinal organoid cultures
Source: Poult Sci. 2026 Jun 24;105(11):107340. doi: 10.1016/j.psj.2026.107340 (PMC13589147; doi:10.1016/j.psj.2026.107340)
Supplement: Supplementary file 1 [file mmc2.docx]

Supplementary figure S1

Leader peptide from human Oncostatin M with Chicken Nrg1 peptide

MGVLLTQRTLLSLVLALLFPSMASMSHLTKCDIKQKAFCVNGGECYMVKDLPNPPRYLCRCPNEFTGDRCQNYVMASFYKHLGIEF MEAE
